# Supplementary material for: Multiomics study of nonalcoholic fatty liver disease
Source: Nat Genet. 2022 Oct 24;54(11):1652–63. doi: 10.1038/s41588-022-01199-5 (PMC9649432; doi:10.1038/s41588-022-01199-5)
Supplement: Supplementary file 1 — Supplementary Figs. 1–15 and Supplementary Note. [file 41588_2022_1199_MOESM1_ESM.pdf]

nature genetics

Article https://doi.org/10.1038/s41588-022-01199-5

Multiomics study of nonalcoholic fatty liver disease

In the format provided by the authors and unedited

Supplementary Material

Supplementary Figure 1: Boxplots showing PDFF for Fatty change of liver (NAFL) (IDEAL n=270, GRE n=97), cirrhosis (IDEAL n=40, GRE n=25), cholangitis (IDEAL n=26, GRE n=19), alcoholic hepatitis (IDEAL n=3, GRE n=3), alcoholic cirrhosis (IDEAL n=7, GRE n=2), alcoholic hepatic failure (IDEAL n=0, GRE n=1), alcoholic fatty liver (IDEAL n=20, GRE n=5), unspecified liver disease (IDEAL n=9, GRE n=4), toxic liver disease (IDEAL n=5, GRE n=2), liver cell carcinoma (IDEAL n=3, GRE n=4), primary biliary cirrhosis (IDEAL n=9, GRE n=5), iron metabolism disorder (IDEAL n=61, GRE n=17), autoimmune hepatitis (IDEAL n=16, GRE n=5), chronic viral hepatitis (IDEAL n=22, GRE n=8), and others (IDEAL n=27,199, GRE n=8251) for the IDEAL and GRE cohorts. Boxplots show the median value (central line) +/- interquartile range (box edges), 95% range (lines), and other values as dots.

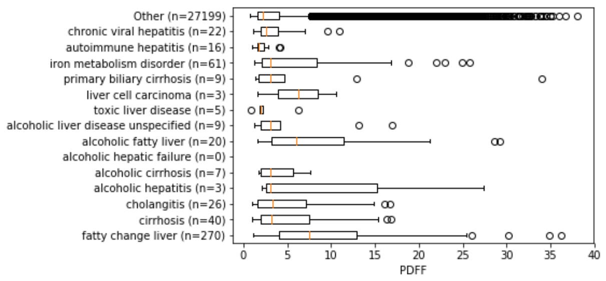

(a) IDEAL cohort

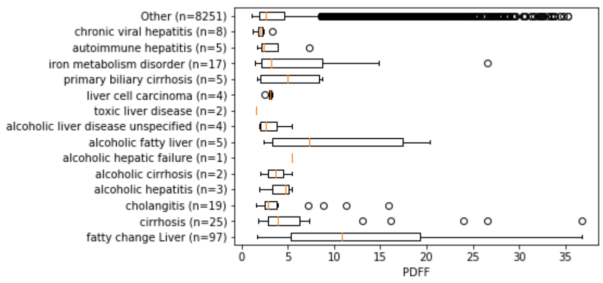

(b) GRE cohort

Supplementary Figure 2: PDFF UK Biobank scores (calculated by others) vs GRE PDFF. The red line is the identity line and blue line the slope from linear regression. N PDFF measurements used = 3869.

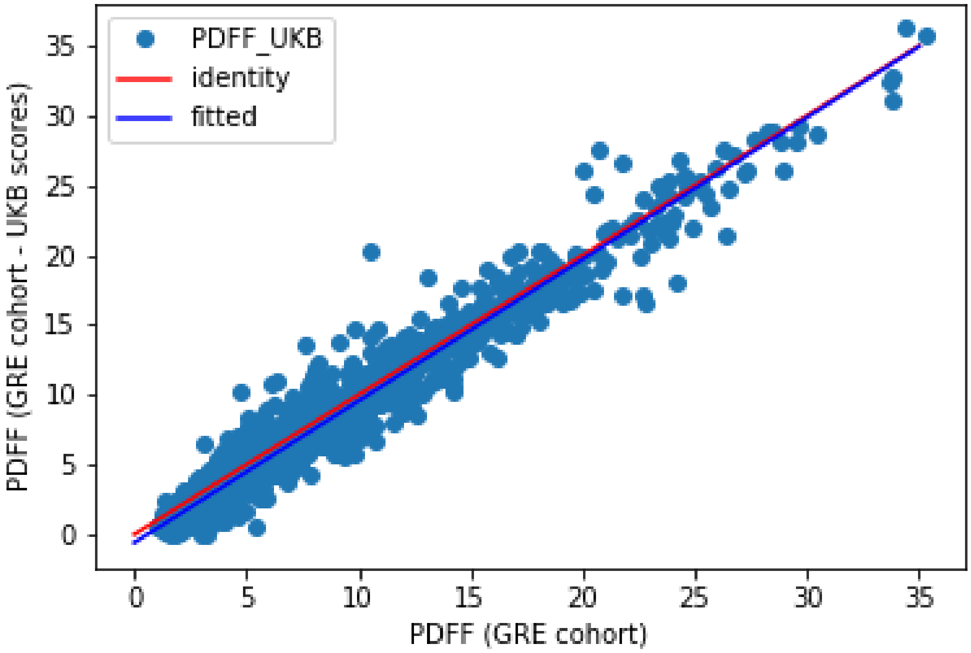

Supplementary Figure 3

a) RNA-sequencing alignment coverage of *MARC1*. The coverage is plotted for carriers and non-carriers of the rare pLOF variant rs772019099 (P.Arg305Ter). There is not a significant difference in *MARC1* expression (P=0.068) between carriers and non-carriers. P-value is two-sided, has not been adjusted for multiple comparisons and is from a linear regression model.

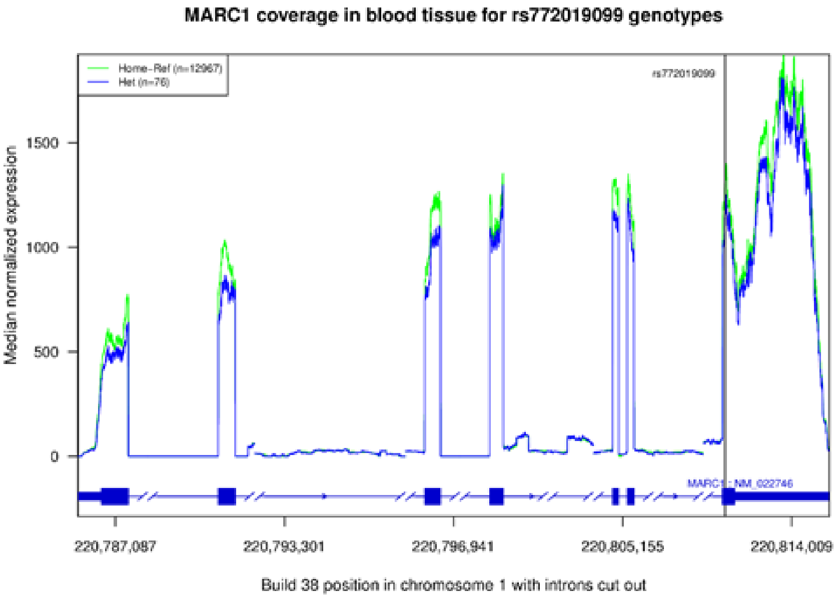

b) : Schematics for canonical isoform (top) and cryptic splice isoform (bottom). Second Supplementary Figurehows percentage splice in (PSI) stratified by genotypes of rs7029757 variant of whole-blood RNAseq individuals. PSI was calculated from RNA-seq reads overlapping exon-exon boundaries.

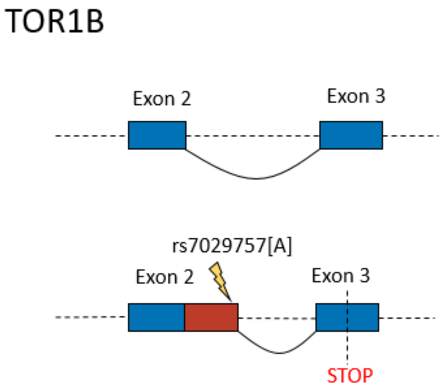

rs7029757 in *TOR1B* generates a cryptic splice site, elongating exon 2 by 50bp leading to a frameshift and introduces a pre-mature stop codon in exon 3 at position 129807219. Elongates exon 2 by 16 amino acids and introduces a stop codon 10 amino acids into exon 3. The variant is located 2bp upstream of cryptic splice site. The variant also associates with decreased expression in lungs, pancreas, cell cultured fibroblasts and esophagus mucosa as top-eQTL. Rs7029757 is the top-eQTL for these tissues in the sense that it shows the strongest association at the locus with expression levels of *TOR1B*. For whole blood rs7029757 is not a top-eQTL since another uncorrelated variant associates more strongly with *TOR1B* expression in blood. Rs7029757, however, associates with expression levels in blood after adjusting for stronger associating variants (-log10(P)=499.9, effect = -0.92 SD), decreasing *TOR1B* transcript abundance. This is consistent with the splice effect observed of the canonical splice site (between exon 2 and 3) and eQTL and sQTL results are therefore consistent in whole blood.

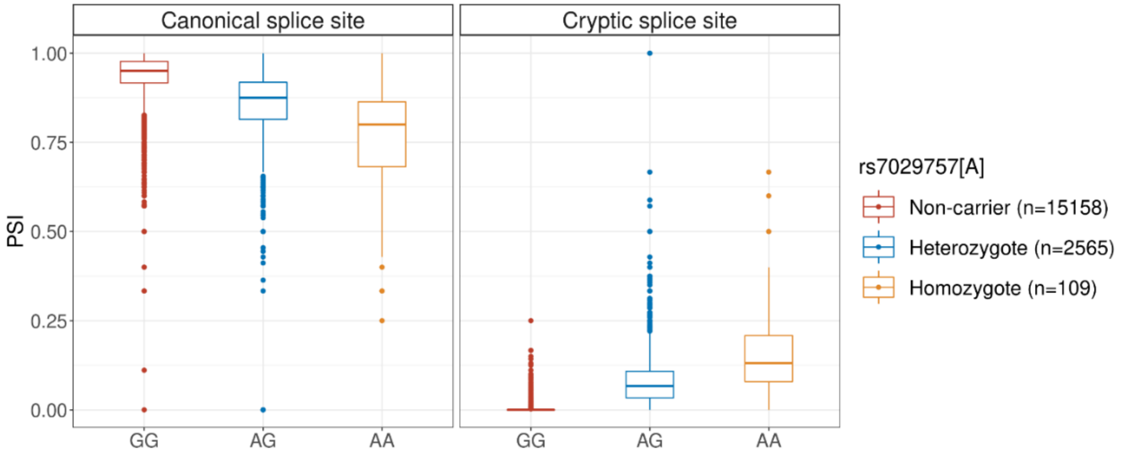

Supplementary Figure 4 - The effects of NAFL variants on the risk of having a diagnose of alcohol liver disease (ALD) compared to the risk of having a NAFL diagnose (ICD code). The red line is the identity line y=x. The effects are show for the allele that has a positive effect on ALD. N_{cases}=3,818 for ALD and N_{cases}=9491. The error bars represent 95% confidence intervals for the effects. Boxplots show the median value (central line) +/- interquartile range (box edges), 95% range (lines), and other values as dots.

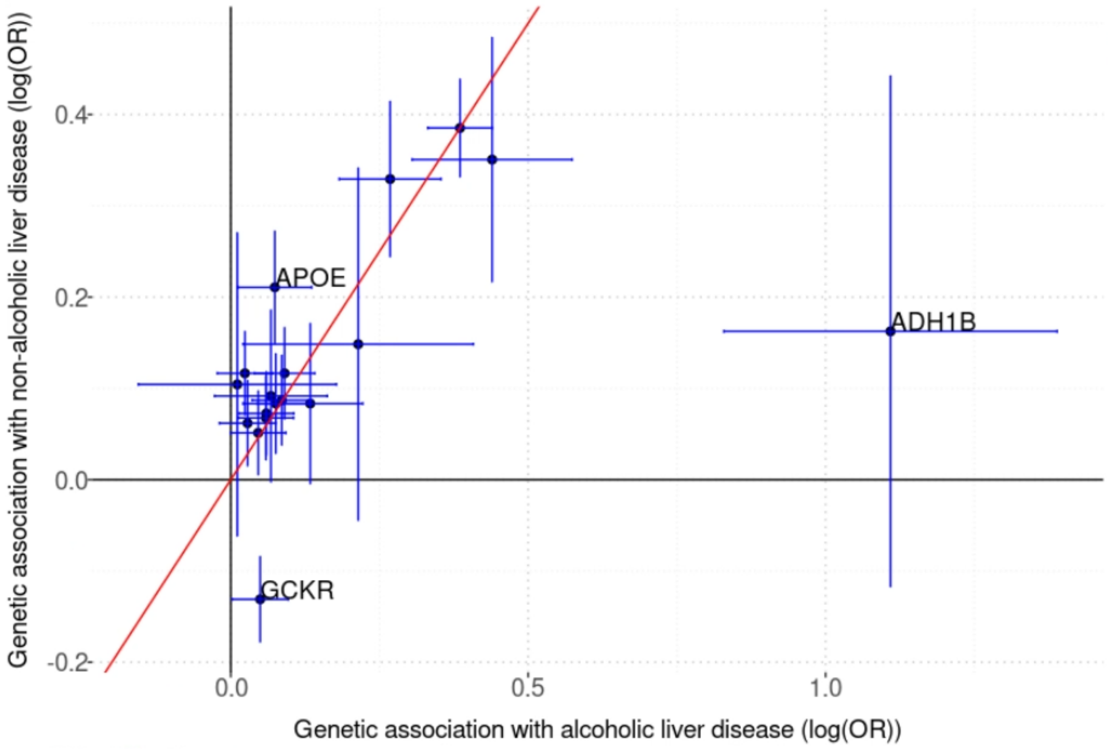

Supplementary Figure 5 Variant x variant interaction on aspartate transaminase (AST). AST was standardized to a normal distribution. The crosses represent mean values in the corresponding strata.

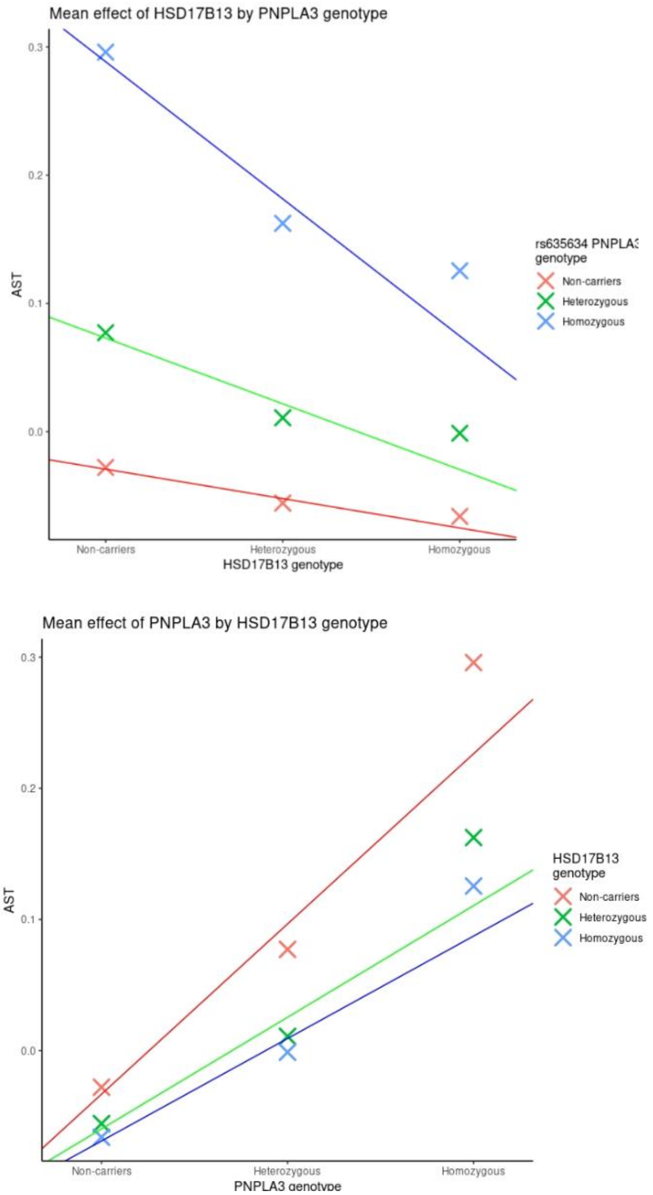

Supplementary Figure 6 Interaction of variants with BMI. The logarithm of PDFF is shown on the y-axis and BMI on the x-axis**.** N PDFF measures = 36116. The numbers, 0 for non-carriers (blue), 1 for heterozygotes (green) and 2 for homozygotes (red) represent mean values per bin and the error bars represent 95% confidence intervals for the mean.

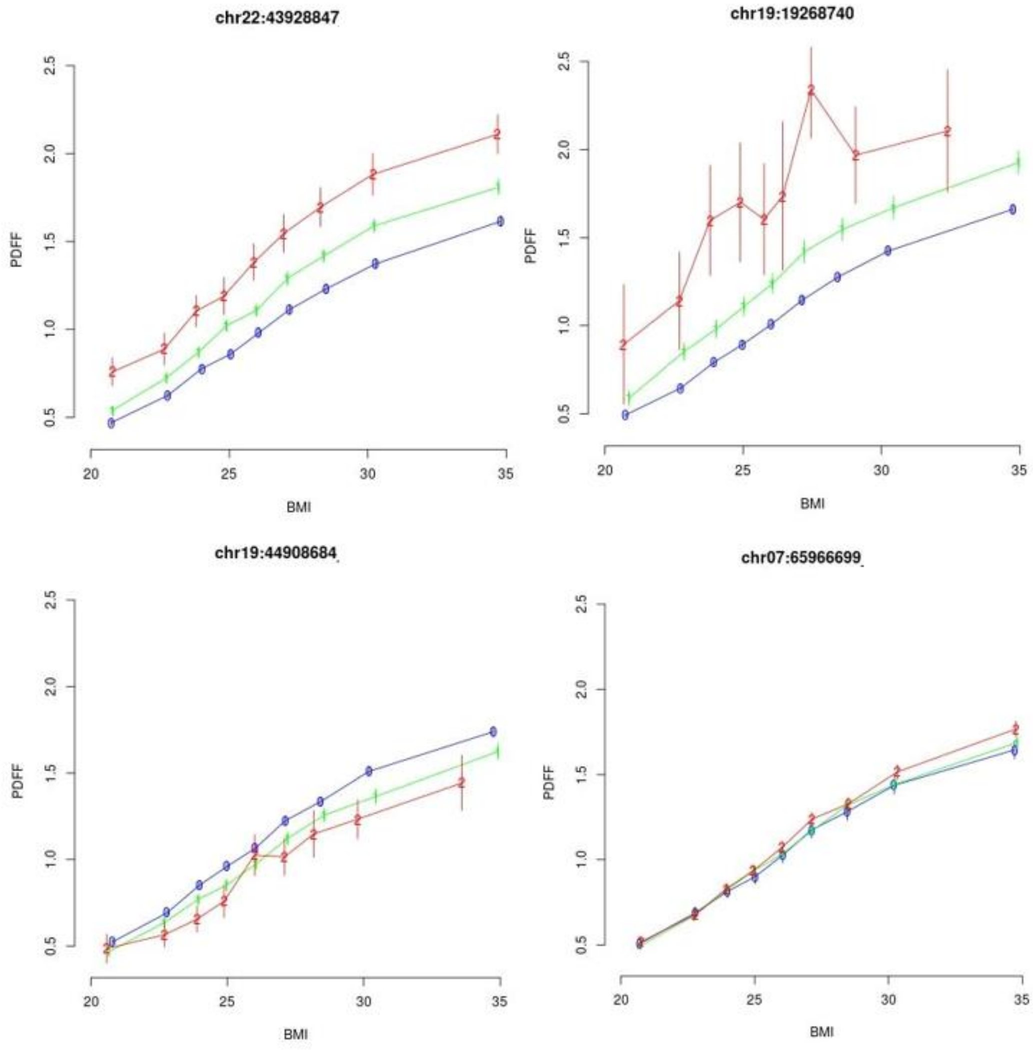

Supplementary Figure 7 shows the relationship between PDFF and age (upper figure) and PDFF and BMI (lower figure), stratified by carrier status of p.Ile148Met in *PNPLA3*. N PDFF measures = 36116. The numbers, 0 for non-carriers (blue), 1 for heterozygotes (green) and 2 for homozygotes (red) represent mean values per bin and the error bars represent 95% confidence intervals for the mean.

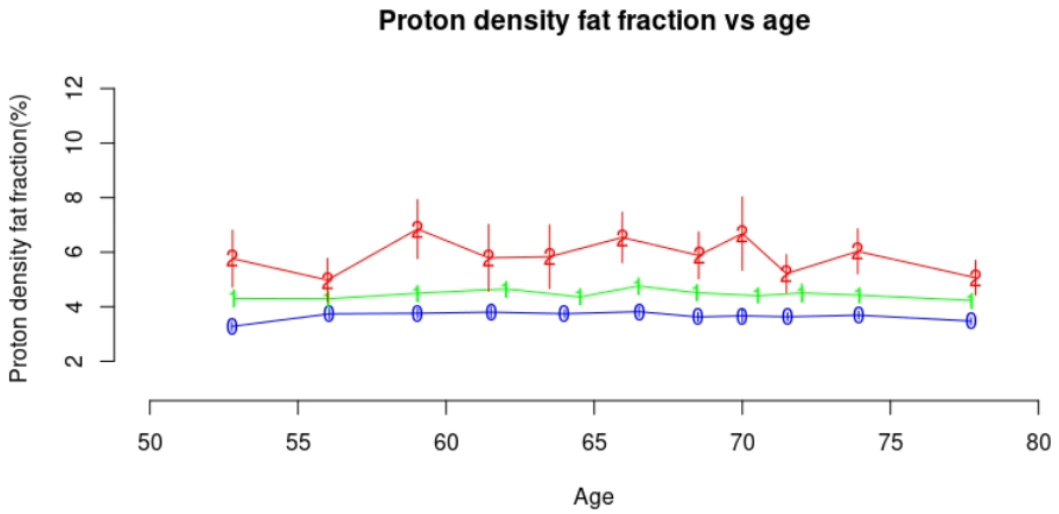

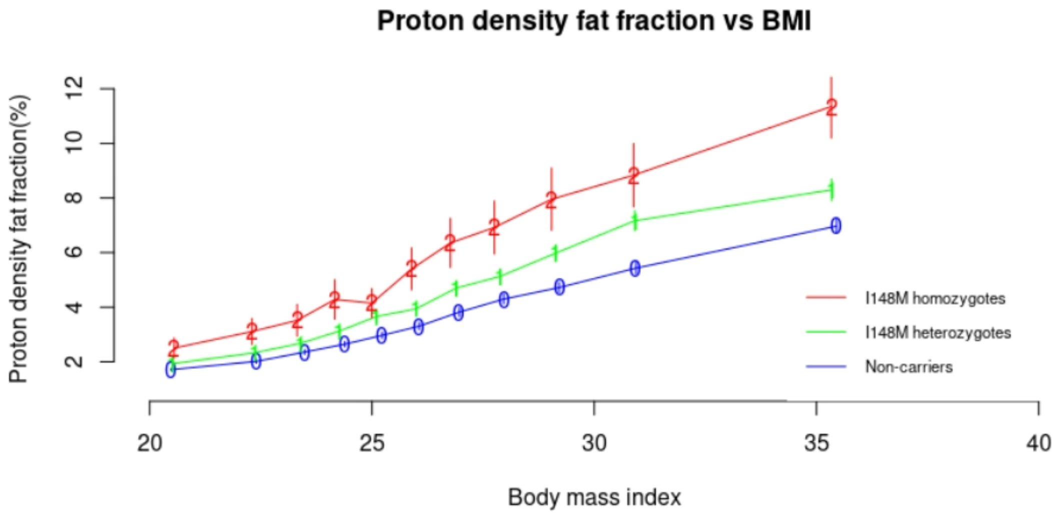

Supplementary Figure 8 Scatter plots comparing BMI and PDFF at first and second imaging. N PDFF measures = 2795.

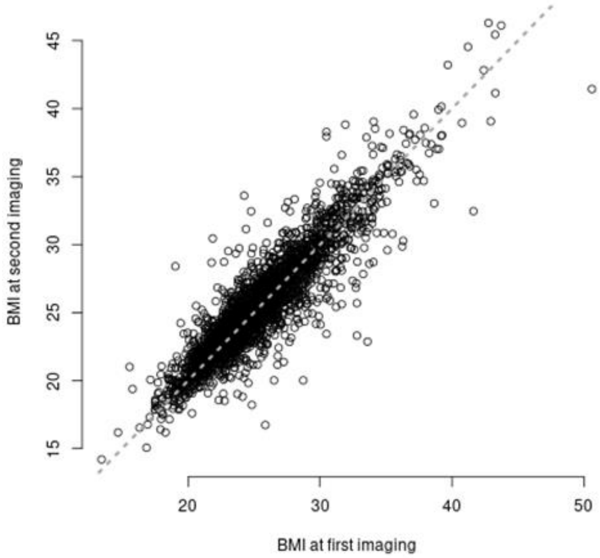

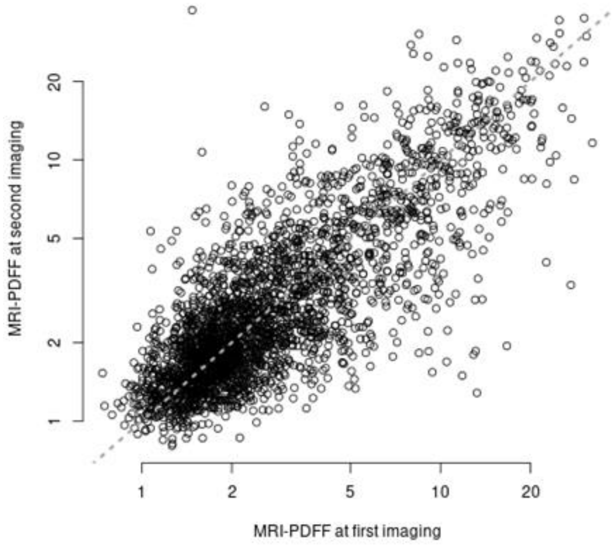

Supplementary Figure 9 Histogram of the change in BMI for indeviduals with 2 PDFF measures. N = 2795.

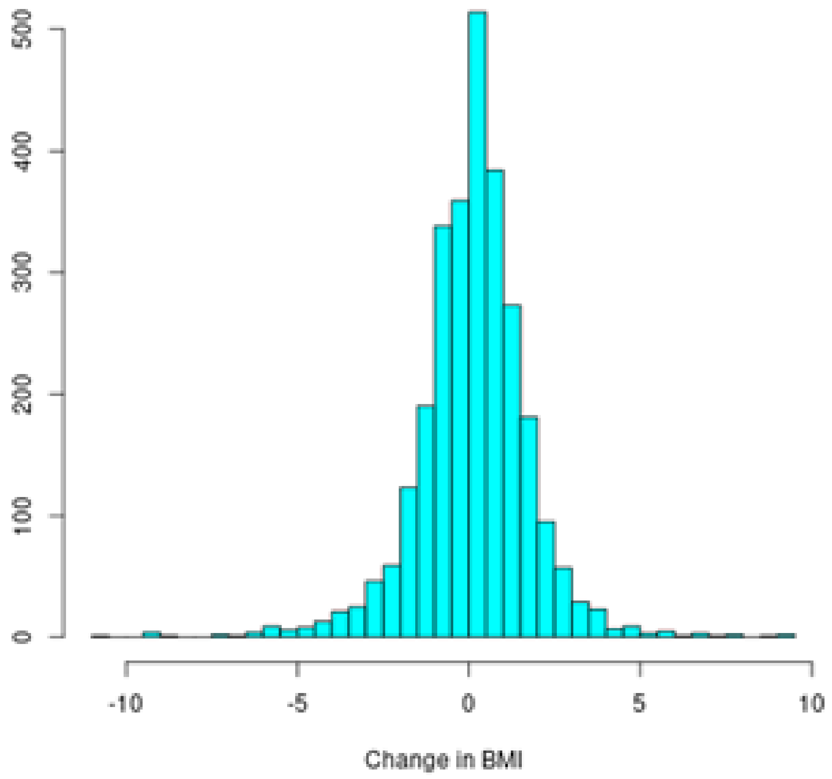

Supplementary Figure 10 Change in BMI shown against change in PDFF. N PDFF measures = 2795. Boxplots show the median value (central line) +/- interquartile range (box edges), 95% range (lines), and other values as dots.

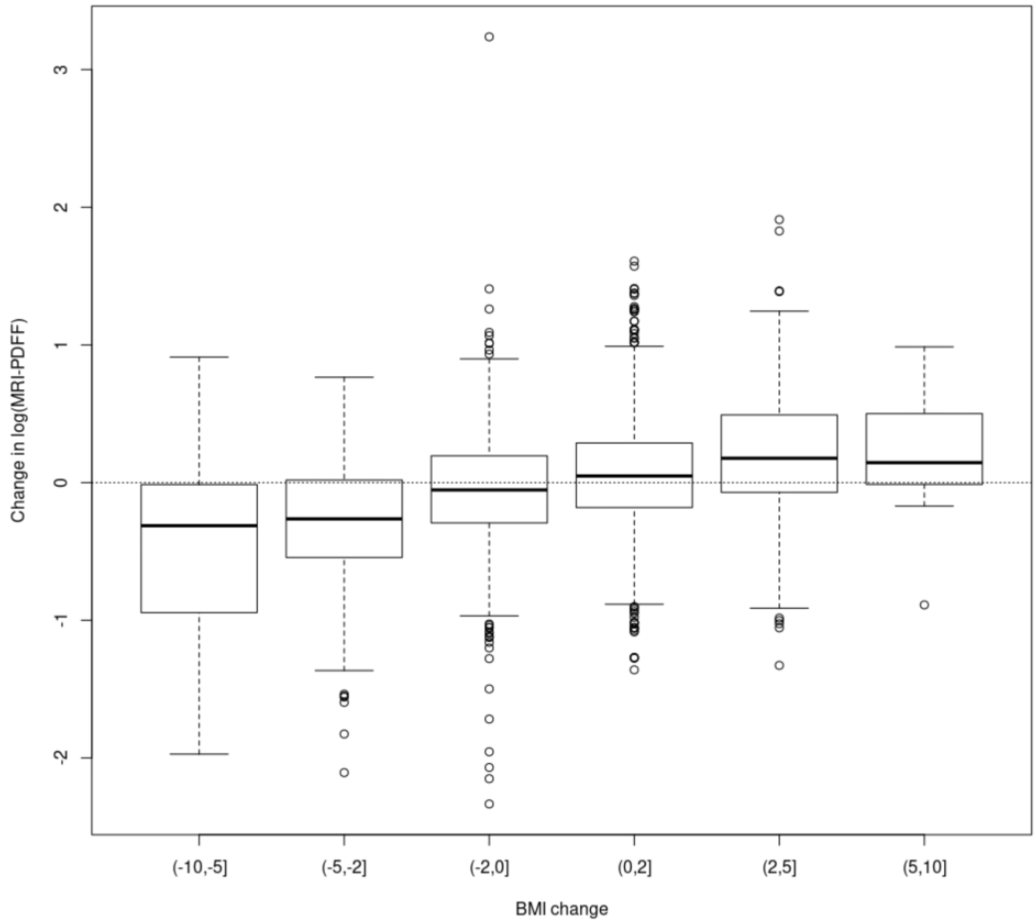

Supplementary Figure 11 Change in BMI compared to change in PDFF stratified by carrier status of p.Ile148Met in *PNPLA3*. N PDFF measures = 2795. The dots represent mean values per bin and the error bars represent 95% confidence intervals for the mean.

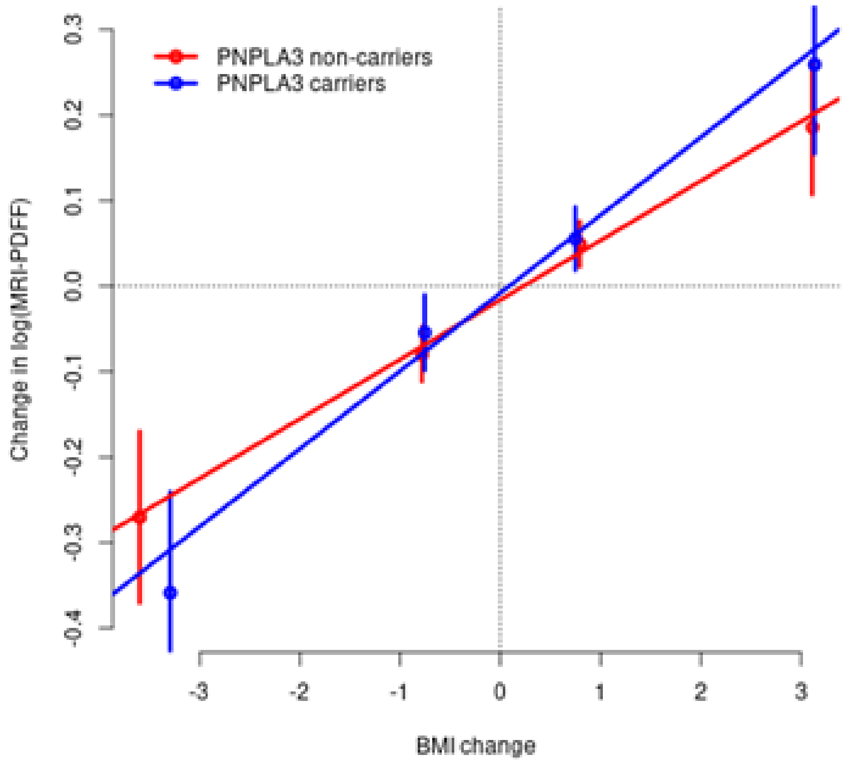

Supplementary Figure 12 Histogram of the change in PDFF stratified by carrier status of p.Ile148Met in *PNPLA3*. N PDFF measures = 2795.

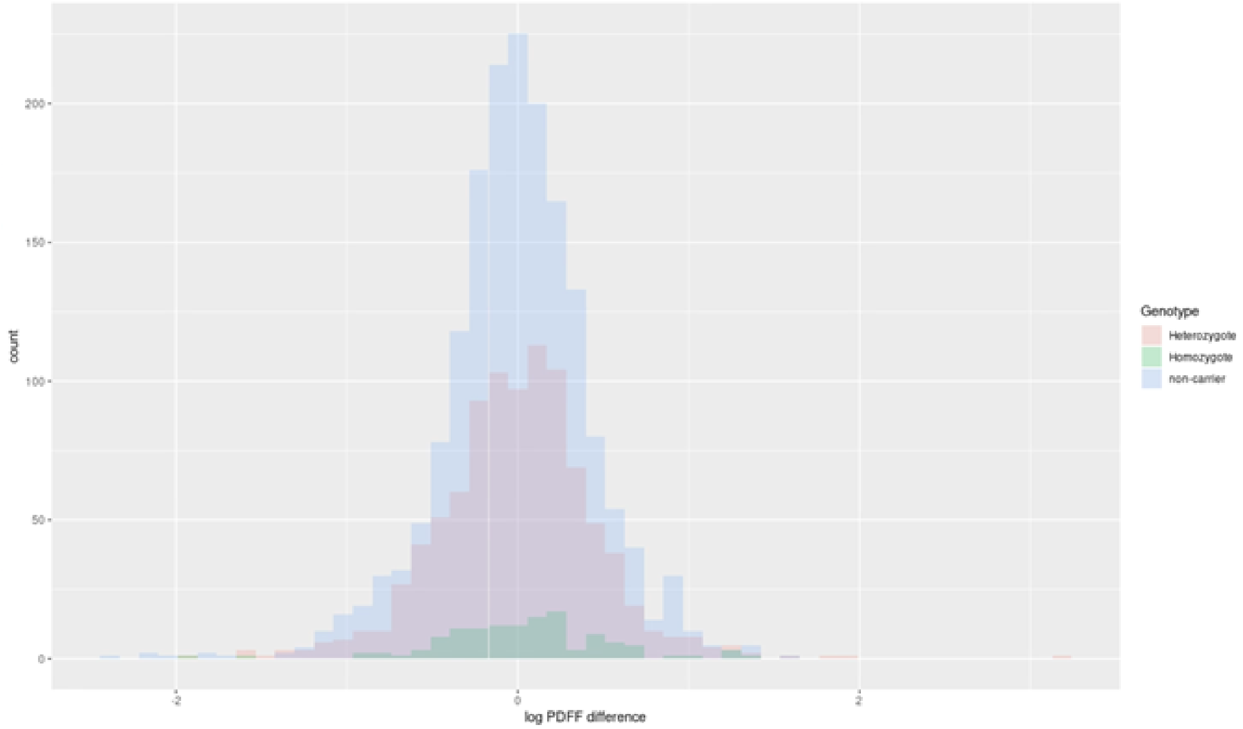

Supplementary Figure 13

a) Scatter plot showing the effect of NAFL variants on PDFF compared to the effects on PLA2G12B levels. The error bars represent 95% confidence intervals for the effects. N PDFF measures = 36116 and N PLA2G12B measures = 35559.

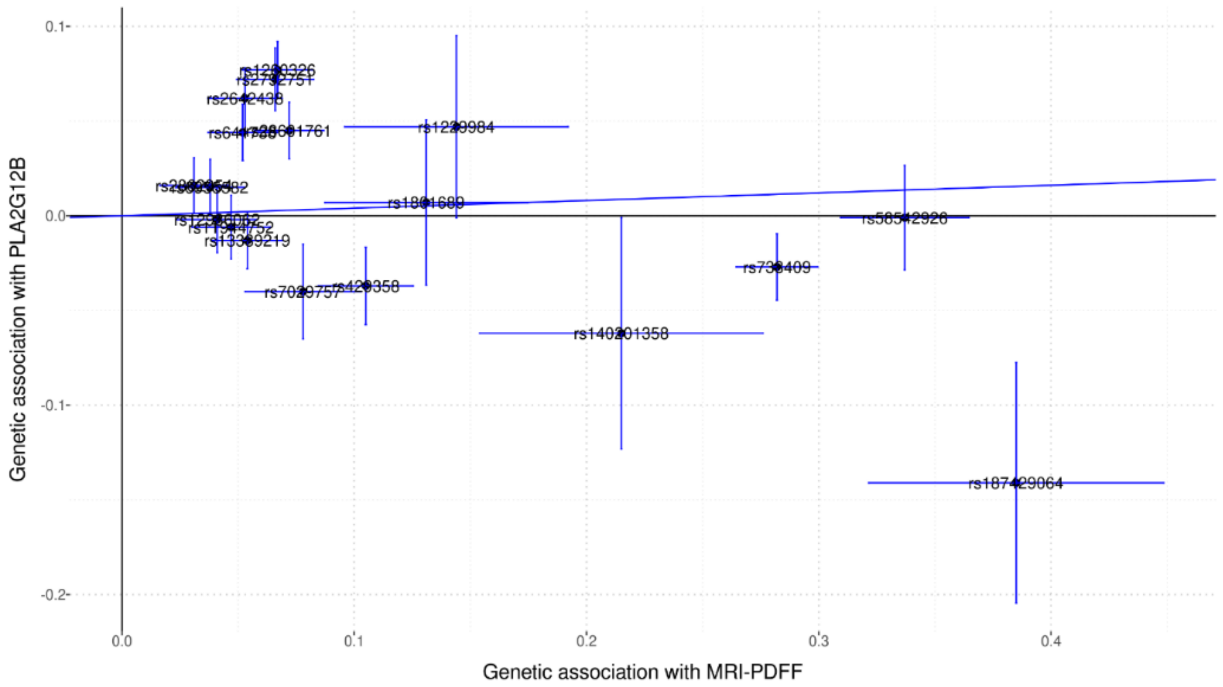

b) Scatter plot showing the effect of TFRC pQTLs (Olink instruments) on TFRC compared to the effects on PDFF. The error bars represent 95% confidence intervals. N PDFF measures = 36116 and N TFRC measures = 47151.

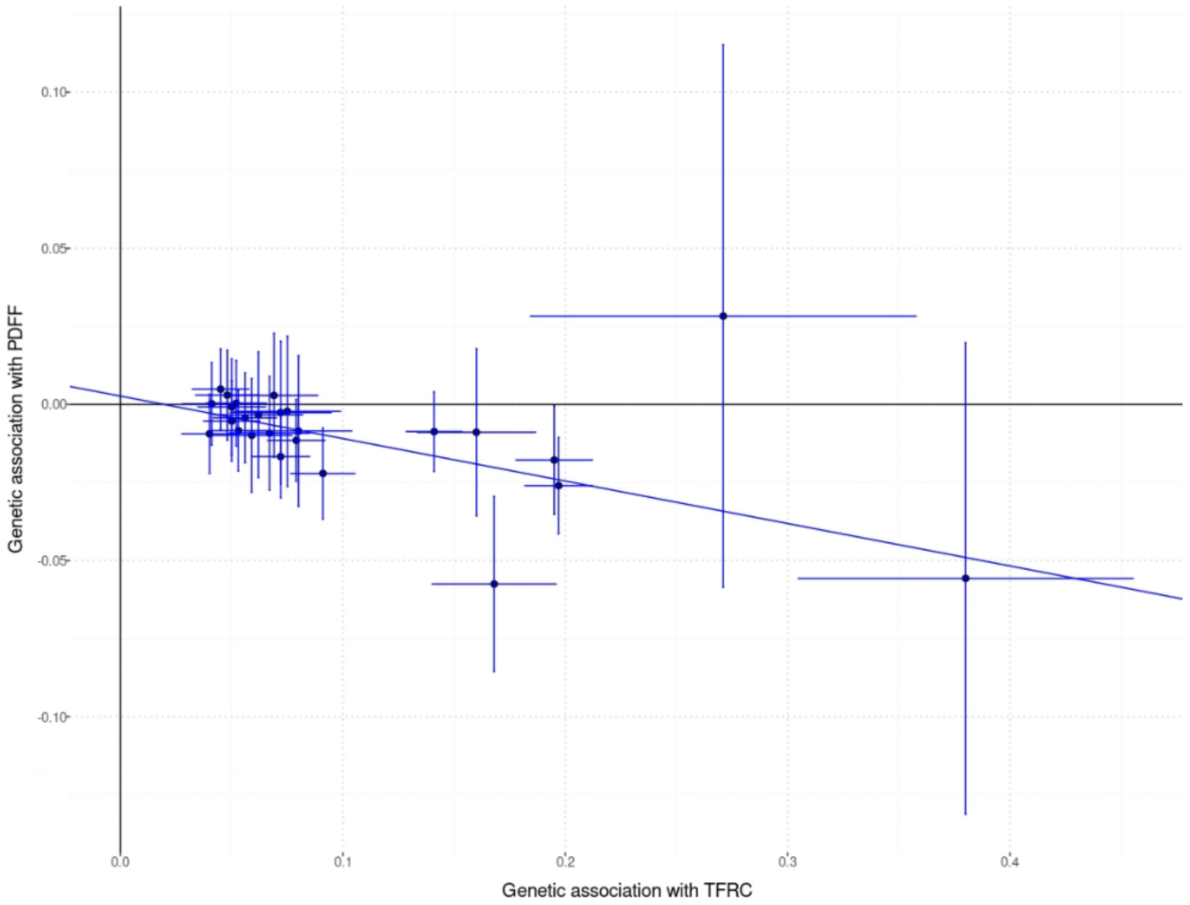

Supplementary Figure 14 Scatter plot showing the effect of proteins on NAFL (y-axis) and cirrhosis (x-axis). The red dots represent the two proteins that are most significantly different in individuals with cirrhosis compared to NAFL.

Somascan:

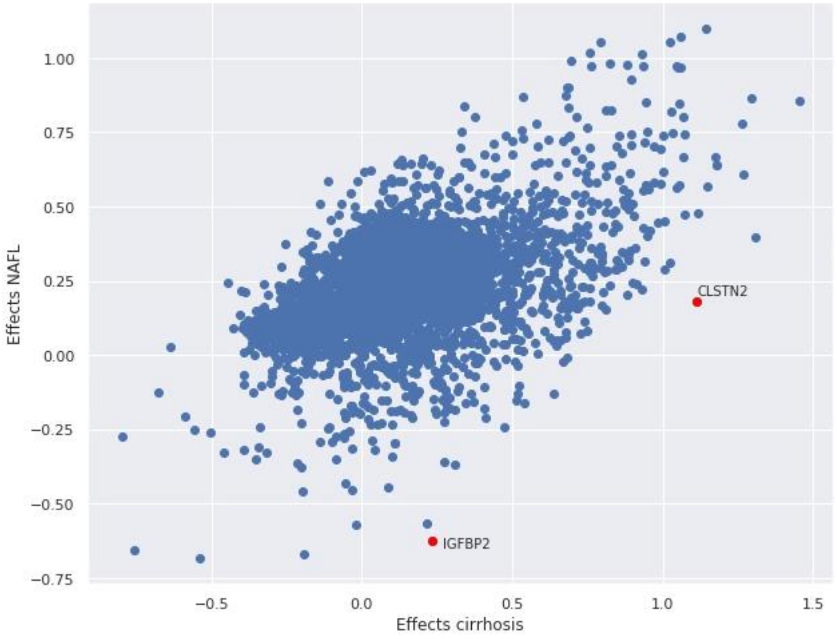

Olink:

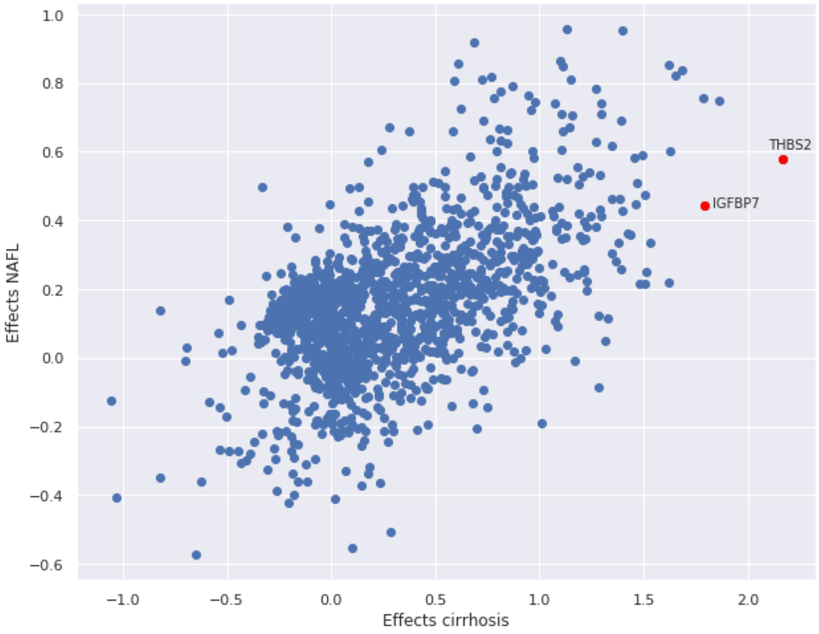

Supplementary Figure 15 shows the effects of the identified variants with 10 proteins. The effect on each protein is shown for the allele that increases PDFF and the risk of NAFL or cirrhosis. Effects are scaled to the range of [-1:1]. Effects are only shown for significant associations after a false discovery rate correction for multiple testing.

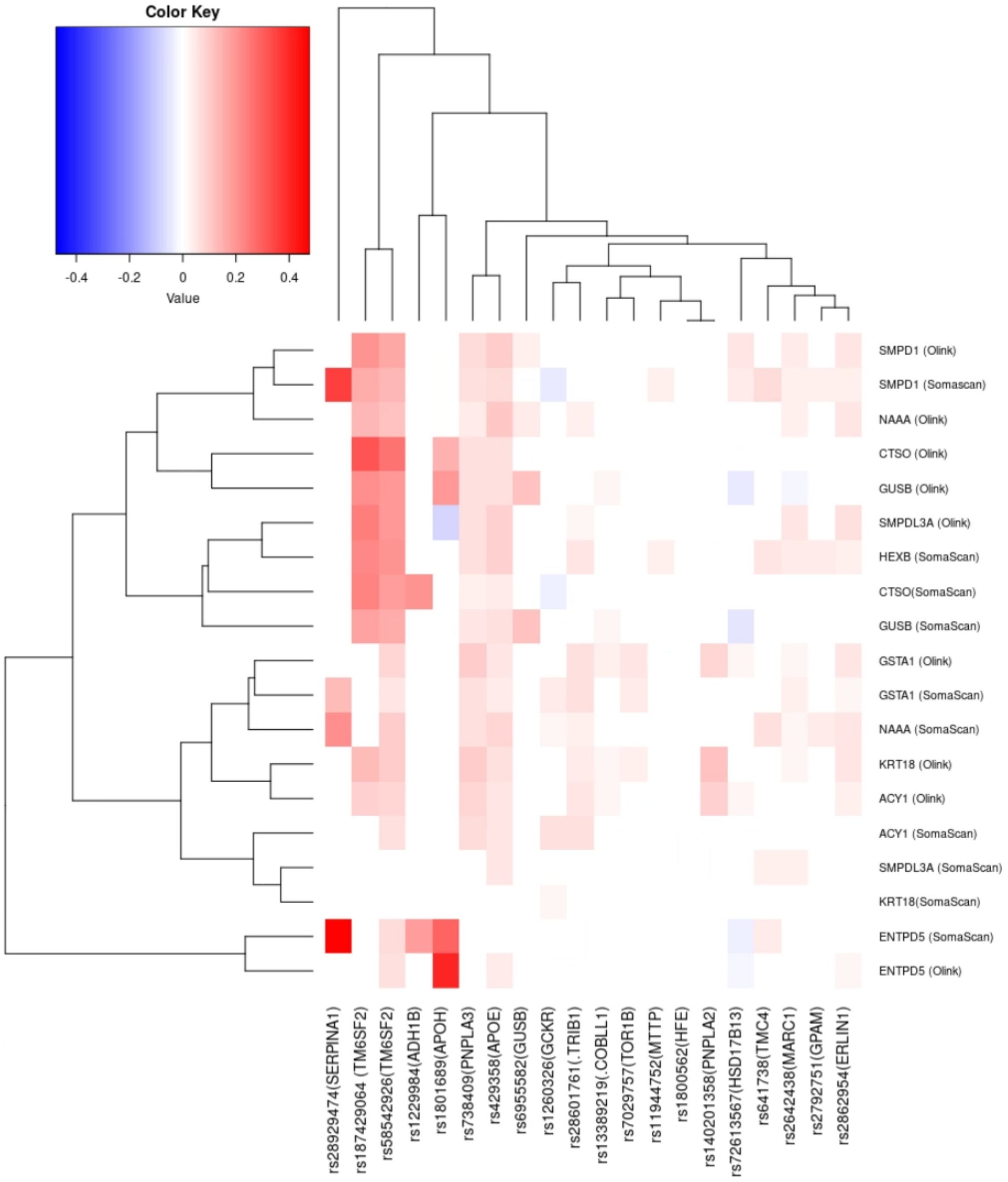

List of members of the DBDS consortium

N	First name	Surname	Email	Affiliation
1	Steffen	Andersen	san.fi@cbs.dk	Department of Finance Copenhagen Business School Copenhagen Denmark
2	Karina	Banasik	karina.banasik@cpr.ku.dk	Novo Nordisk Foundation Center for Protein Research Faculty of Health and Medical Sciences University of Copenhagen Copenhagen Denmark
3	Søren	Brunak	soren.brunak@cpr.ku.dk	Novo Nordisk Foundation Center for Protein Research Faculty of Health and Medical Sciences University of Copenhagen Copenhagen Denmark
4	Kristoffer	Burgdorf	kristoffer.soelvsten.burgdorf@regionh.dk	Department of Clinical Immunology Copenhagen University Hospital Copenhagen Denmark
5	Maria	Didriksen	maria.didriksen@regionh.dk	Department of Clinical Immunology Copenhagen University Hospital Copenhagen Denmark
6	Khoa Manh	Dinh	khoadinh@rm.dk	Department of Clinical Immunology Aarhus University Hospital Aarhus
7	Christian	Erikstrup	christian.erikstrup@skejby.rm.dk	Department of Clinical Immunology Aarhus University Hospital Aarhus Denmark
8	Daniel	Gudbjartsson	Daniel.Gudbjartsson@decode.is	deCODE genetics Reykjavik Iceland
9	Thomas Folkmann	Hansen	thomas.hansen@regionh.dk	Danish Headache Center department of Neurology Rigshospitalet Glostrup
10	Henrik	Hjalgrim	HHJ@ssi.dk	Department of Epidemiology Research Statens Serum Institut Copenhagen Denmark
11	Gregor	Jemec	gbj@regionsjaelland.dk	Department of Clinical Medicine Sealand University hospital Roskilde Denmark
12	Poul	Jennum	poul.joergen.jennum@regionh.dk	Department of clinical neurophysiology at University of Copenhagen Copenhagen Denmark
14	Pär Ingemar	Johansson	Per.Johansson@regionh.dk	Department of Clinical Immunology, Copenhagen University Hospital Copenhagen Denmark
15	Margit Anita Hørup	Larsen	Margit.Anita.Hoerup.Larsen@regionh.dk	Department of Clinical Immunology Copenhagen University Hospital Copenhagen Denmark
16	Susan	Mikkelsen	susanmke@rm.dk	Department of Clinical Immunology Aarhus University Hospital Aarhus
17	Kasper Rene	Nielsen	k.nielsen@rn.dk	Department of Clinical Immunology Aalborg University Hospital Aalborg Denmark
18	Mette	Nyegaard	nyegaard@biomed.au.dk	Department of Biomedicine Aarhus University Denmark
19	Sisse Rye	Ostrowski	sisse.rye.ostrowski@regionh.dk	Department of Clinical Immunology Copenhagen University Hospital Copenhagen Denmark
20	Ole Birger	Pedersen	olbp@regionsjaelland.dk	Department of Clinical Immunology Zealand University Hospital, Køge
21	Kari	Stefansson	kari.stefansson@decode.is	deCODE genetics Reykjavik Iceland
22	Hreinn	Stefánsson	hreinn.stefansson@decode.is	deCODE genetics Reykjavik Iceland
23	Susanne	Sækmose	sugs@regionsjaelland.dk	Department of Clinical Immunology Zealand University Hospital, Køge
24	Erik	Sørensen	Erik.Soerensen@regionh.dk	Department of Clinical Immunology Copenhagen University Hospital Copenhagen Denmark
25	Unnur	Þorsteinsdóttir	Unnur.Thorsteinsdottir@decode.is	deCODE genetics Reykjavik Iceland
26	Mie	Topholm Brun	mie.topholm.bruun@rsyd.dk	Department of Clinical Immunology Odense University Hospital Odense Denmark
27	Henrik	Ullum	Henrik.Ullum@regionh.dk	Department of Clinical Immunology Copenhagen University Hospital Copenhagen Denmark
28	Thomas	Werge	thomas.werge@regionh.dk	Institute of Biological Psychiatry Mental Health Centre Sct. Hans Copenhagen University Hospital Roskilde Denmark
