# Supplementary material for: Multiomics study of nonalcoholic fatty liver disease
Source: Nat Genet. 2022 Oct 24;54(11):1652–63. doi: 10.1038/s41588-022-01199-5 (PMC9649432; doi:10.1038/s41588-022-01199-5)
Supplement: Supplementary file 2 — Reporting Summary [file 41588_2022_1199_MOESM2_ESM.pdf]

nature portfolio

Corresponding author(s): Gardar Sveinbjornsson, Kari Stefansson

Last updated by author(s): Jun 28, 2022

Reporting Summary

Nature Portfolio wishes to improve the reproducibility of the work that we publish. This form provides structure for consistency and transparency in reporting. For further information on Nature Portfolio policies, see our Editorial Policies and the Editorial Policy Checklist.

Statistics

For all statistical analyses, confirm that the following items are present in the figure legend, table legend, main text, or Methods section.

n/a	Confirmed	
☐	☒	The exact sample size (*n*) for each experimental group/condition, given as a discrete number and unit of measurement
☐	☒	A statement on whether measurements were taken from distinct samples or whether the same sample was measured repeatedly
☐	☒	The statistical test(s) used AND whether they are one- or two-sided<br>*Only common tests should be described solely by name; describe more complex techniques in the Methods section.*
☐	☒	A description of all covariates tested
☐	☒	A description of any assumptions or corrections, such as tests of normality and adjustment for multiple comparisons
☐	☒	A full description of the statistical parameters including central tendency (e.g. means) or other basic estimates (e.g. regression coefficient) AND variation (e.g. standard deviation) or associated estimates of uncertainty (e.g. confidence intervals)
☐	☒	For null hypothesis testing, the test statistic (e.g. *F*, *t*, *r*) with confidence intervals, effect sizes, degrees of freedom and *P* value noted<br>*Give P values as exact values whenever suitable.*
☒	☐	For Bayesian analysis, information on the choice of priors and Markov chain Monte Carlo settings
☒	☐	For hierarchical and complex designs, identification of the appropriate level for tests and full reporting of outcomes
☒	☐	Estimates of effect sizes (e.g. Cohen's *d*, Pearson's *r*), indicating how they were calculated

Our web collection on statistics for biologists contains articles on many of the points above.

Software and code

Policy information about availability of computer code

Data collection	No software was used for data collection.
Data analysis	We used publicly available software in conjunction with methods developed at deCODE Genetics as described in the methods section.<br><br>Graphtyper version 2, https://github.com/DecodeGenetics/graphtyper;<br>We used R, version 3.6.0 and Python, version 3.9 extensively to analyze data and create plots;<br>Multi-trait analysis of genome-wide association summary statistics version 1, https://github.com/JonJala/mtag;<br>PANTHER v.16.0, http://www.pantherdb.org/tools/;<br>Variant Effect Predictor (release 100), https://github.com/Ensembl/ensembl-vep;<br>BOLT-LMM version 2.1, https://data.broadinstitute.org/alkesgroup/BOLT-LMM/downloads/;<br>IMPUTE2 version 2.3.1, https://mathgen.stats.ox.ac.uk/impute/impute_v2.html;<br>dbSNP version 140, http://www.ncbi.nlm.nih.gov/SNP/;<br>STAR software package, version 2.7.10, https://github.com/alexdobin/STAR;<br>Ensembl version 87, https://www.ensembl.org/index.html;<br>LeafCutter version 1 , https://github.com/davidaknowles/leafcutter;<br>kallisto version 0.46, https://github.com/pachterlab/kallisto

For manuscripts utilizing custom algorithms or software that are central to the research but not yet described in published literature, software must be made available to editors and reviewers. We strongly encourage code deposition in a community repository (e.g. GitHub). See the Nature Portfolio guidelines for submitting code & software for further information.

Data

Policy information about availability of data

All manuscripts must include a data availability statement. This statement should provide the following information, where applicable:

- Accession codes, unique identifiers, or web links for publicly available datasets
- A description of any restrictions on data availability
- For clinical datasets or third party data, please ensure that the statement adheres to our policy

GWAS summary statistics for PDFF, NAFL, cirrhosis and HCC are available at https://www.decode.com/summarydata/. Sequence variants tested for association have been deposited in the European Variation Archive under accession number PRJEB15197 (https://www.ebi.ac.uk/ena/browser/view/PRJEB15197). FinnGen data are publicly available and were downloaded from https://finngen.fi/. The UKB data was downloaded under application number 56270. Proteomics data and protein mapping to UniProt identifiers and gene names were provided by SomaLogic and Olink. Other data and code presented in this study are included in this publication and its Supplementary information.

Field-specific reporting

Please select the one below that is the best fit for your research. If you are not sure, read the appropriate sections before making your selection.

☒ Life sciences ☐ Behavioural & social sciences ☐ Ecological, evolutionary & environmental sciences

For a reference copy of the document with all sections, see nature.com/documents/nr-reporting-summary-flat.pdf

Life sciences study design

All studies must disclose on these points even when the disclosure is negative.

Sample size	Sample sizes are reported in the article and correspond to all available data
Data exclusions	No available data was excluded from the study
Replication	The NAFLD GWAS analysis was performed using data from 4 populations (Iceland, UK, USA and Finland) and results across populations were compared.
Randomization	Not applicatble (GWAS study, not a randomized trial)
Blinding	Not applicable (GWAS study, not a randomized trial, so no blinding is required)

Reporting for specific materials, systems and methods

We require information from authors about some types of materials, experimental systems and methods used in many studies. Here, indicate whether each material, system or method listed is relevant to your study. If you are not sure if a list item applies to your research, read the appropriate section before selecting a response.

Materials & experimental systems

n/a	Involved in the study	
☒	☐	Antibodies
☒	☐	Eukaryotic cell lines
☒	☐	Palaeontology and archaeology
☒	☐	Animals and other organisms
☐	☒	Human research participants
☒	☐	Clinical data
☒	☐	Dual use research of concern

Methods

n/a	Involved in the study	
☒	☐	ChIP-seq
☒	☐	Flow cytometry
☒	☐	MRI-based neuroimaging

Human research participants

Policy information about studies involving human research participants

Population characteristics	A detailed description of population characteristics can be found in the methods section.<br>The UK Biobank project is a large prospective cohort study of ~500,000 individuals from across the United Kingdom, aged between 40-69 years at recruitment. In the UK Biobank 46% recruited were male, 54% female. 57% aged 40-59 years; 43% aged 60-69 years.<br>The Icelandic deCODE genetics study is based on whole-genome sequence data from 49,708 Icelanders participating in various research projects at deCODE genetics. Variants identified through whole-genome sequencing were imputed into

155,250 chip-genotyped Icelanders as well as their untyped close relatives based on genealogy.
Finngen summary statistics, including fatty liver disease and cirrhosis, were imported on December 2020 from a source available to researchers (version 4; https://www.finngen.fi/en/access_results) and methods have been documented (https://finngen.gitbook.io/documentation/).
The Copenhagen Hospital Biobank Cardiovascular Study (CHB- CVDC) was used to acquire secondary cardiovascular phenotypes. CHB-CVDC involves a targeted selection of patients with cardiovascular disease from the CHB, a biobank based on patient blood samples drawn in Danish hospitals. For binary phenotypes, the control group included blood donors from The Danish Blood Donor Study (DBDS).
The samples from the US (Intermountain dataset) were WGS using NovaSeq Illumina technology and genotyped using Illumina GSA chips.

Recruitment

For the deCODE Genetics study individuals were recruited through various research projects at deCODE genetics. The participants are a large fraction of the adult Icelandic population.
UK Biobank holds data on half a million participants throughout the UK. All participants in UK Biobank were recruited through assessment centres, designed specifically for this purpose.
The US data are individuals recruited at the Intermountain healthcare.
The FinnGen database consists of samples collected from Finnish biobanks.
CHB-CVDC involves a targeted selection of patients with cardiovascular disease from the CHB, a biobank based on patient blood samples drawn in Danish hospitals.

Ethics oversight

All participating subjects in the deCODE genetics study who donated blood signed informed consent. The personal identities of the participants and biological samples were encrypted by a third-party system. The study was approved by the Icelandic Data Protection Authority and the National Bioethics Committee of Iceland (no VSN-20-182).
The CHB-CVDC has been approved by The National Committee on Health Research Ethics (1708829) and the Danish Data Protection Agency (P-2019-93). The Danish Blood Donor Study (DBDS), approved by the Danish Data Protection Agency (P-2019-99) and the Scientific Ethical Committee system (NVC 1700407).
The FinnGen database consists of samples collected from the Finnish biobanks and and phenotype data collected at the national health registers. The Coordinating Ethics Committee of the Helsinki and Uusimaa Hospital District evaluated and approved the FinnGen research project. The project complies with existing legislation (in particular the Biobank Law and the Personal Data Act). The official data controller of the study is University of Helsinki.
The UK Biobank Resource was used under application number 56270. All phenotype and genotype data were collected following an informed consent obtained from all participants. The North West Research Ethics Committee reviewed and approved UK Biobank's scientific protocol and operational procedures (REC Reference Number: 06/MRE08/65).
For the Intermountain dataset, the Intermountain Healthcare Institutional Review Board approved the study, and all participants provided written informed consent prior to enrollment.

Note that full information on the approval of the study protocol must also be provided in the manuscript.
